# Scent and the Cinema

**DOI:** 10.1177/2041669520969710

**Published:** 2020-11-25

**Authors:** Charles Spence

**Affiliations:** Crossmodal Research Laboratory, Oxford University, Oxford, United Kingdom

**Keywords:** scent, cinema, entertainment, Perolin, Feelies

## Abstract

From the very earliest days of public cinema (moving pictures), there has been consideration about how odors and scents might influence the viewer’s experience. While initially this was primarily a concern with how to eliminate the malodor of the cinema-goers themselves, in more recent times, there have been a number of well-publicized attempts to add synchronized pleasant (and, on occasion, also unpleasant) scents to “enhance” the cinema experience. While early solutions such as *AromaRama* and *Smell-O-Vision* were beset by technical challenges, low-tech scratch and sniff (*Odorama*) and, more recently, Edible Cinema-type solutions (where the audience get to consume flavourful, and often aromatic, morsels in time with the events on screen) have proved somewhat more successful. Nevertheless, there are a number of key psychological factors that will likely inhibit the uptake of scented cinema in the future, even should the technical and financial issues (associated with retrofitting cinemas, and providing the appropriate fragrances) one day be satisfactorily resolved. These include the phenomenon of “inattentional anosmia” as well as the “fundamental misattribution error,” whereby people (who are, by-and-large, visually-dominant) tend to attribute their enjoyment to the action seen on screen, rather than to smell, and hence are unlikely to pay a premium for the latter.

## Introduction

The artistic use of scent and fragrance has a long history in theatrical and, to a lesser extent, operatic productions (see Banes, 2001; Banes & Lepecki, 2007), as well as, on occasion, musical productions (e.g., Macdonald, 1983; Sebag-Montefiore, 2016). There has also been growing interest in the use of scent to augment the displays in museums and art galleries (see Spence, 2020a, for a recent review). In the contemporary era, much of this interest has been spurred on by attempts to enhance the multisensory experience by means of engaging more of the audience’s, or visitors’, senses (Pulh et al., 2008). In the setting of the cinema, there is also a continuing desire to deliver something more (or different) than can be experienced at home (see also B. Barnes, 2014; Hanson, 2013; Lamont, 2016; Malvern, 2018). In fact, back in the 1950s, cinema owners were already worrying about people’s increasing tendency to stay at home in front of the TV, rather than to go out to the movies (Paterson, 2006). As Kaye (2004) notes: “There was a rush of to create technologies to lure customers back to the cinema: 3-D glasses, vibrating seats, and, of course, scented films” (p. 55).^1^

However, around the turn of the 19th century, the most pressing question for many cinema owners was how to eliminate the smell of the audience themselves who found themselves in the confined spaces, often with poor ventilation, where early films were typically shown (see J. Barnes, 1983). Once the problem of malodorous audiences had been resolved, the time was ripe for the addition of scent to augment the cinematic experience. And, hard though it may be to believe today, the opinion, at least in some quarters, would seem to have been that adding scent to cinema might well help to elevate the movie watching experience in much the same way that adding color to Black and White films was already starting to do (see Bedi, 2017; Frost, 2006; Hanssen, 2006; Misek, 2010).^2^ Note here only the fact that one of the first publicized technologically controlled scented films was shown in the United States in 1940,^3^ the same year as the huge global success of early color movies such as *Gone with the Wind* and *The Wizard of Oz.* Meanwhile, Walt Disney’s enduring favorite *Fantasia* was released the following year. Intriguingly, Disney had apparently toyed, albeit briefly, with the idea of introducing scent to *Fantasia* but was eventually dissuaded by the cost (see Hawking, 2015). According to Canemaker (1988, p. 76), Disney considered flower perfume notes for the *Nutcracker Suite* and *Clair de Lune* (night-blooming cereus), incense for the *Ave Maria* and *Credo*, and gunpowder for the *Sorcerer’s Apprentice* sequence. The latter suggested by Leopold Stokowski who conducted the Philadelphia Orchestra in seven works of classical music visualized by artists and animators in the film. Meanwhile, in 1939, Groucho Marx was quoted as saying: “I'm not really interested in radio, I'm waiting for the smellies or tasties. I want to crash through to the unseen audience in six assorted perfumes or flavors.” (Arce, 1979, p. 262).

Inspired by the Italian Futurists, Aldous Huxley (1932) also mentioned the olfactorily and haptically enhanced cinema of the future in his dystopian novel *Brave New World*. There, the British novelist writes: *COLOURED, STEREOSCOPIC FEELY. WITH SYNCHRONIZED SCENT-ORGAN ACCOMPANIMENT.* “Take hold of those metal knobs on the arms of your chair,” whispered Lenina. “Otherwise you won’t get any of the feely effects” (Huxley, 1932, p. 119; Huxley, 2007, pp. 145–146). The notion of tactile cinema, as captured by Huxley’s “Feelies” has, though, fared little better over the intervening decades (see Frost, 2006; Paterson, 2006) than the “Smellies,” specifically, the idea of scent-enhanced cinema.

However, from the first attempts to add multiple scents to match what was seen on screen, technological challenges have continued to hamper the use of scent in the cinema (see Gilbert, 2008, for an entertaining review). While there have been several patented attempts to bring scent to cinema, the question of what exactly the goal of this form of sensory augmentation actually was has rarely been considered. The use of scent has often been pleonastic (i.e., redundant with what was shown on the stage or screen; Banes, 2001). That said, the more successful use of scent often references both the action/setting seen on stage or screen but also symbolizes something else as well. For example, J. G. Harris (2007) gives the example of how early audiences watching Shakespeare’s Macbeth in the 1600s would likely have understood the pervasive presence of the sulfurous smell of gunpowder (from the squibs—these were fireworks that would have been set off at the start of the play) in the theater, to connect both to on-stage events and to recent political events in England, namely, the Catholic plot of Guy Fawkes to blow up Parliament—the Gunpowder Plot—that would have been current at the time.

## Early History of Malodor in the Cinema

The earliest moving pictures (typically documentaries) were shown on the fairgrounds in the United Kingdom in the 1890s, with one of the main figures responsible being Randall Williams, your author’s great, great grandfather, once known as “The King of Showmen” (see Gashinski, 2011). However, as the projection machinery/technology rapidly developed, it started to become much more cumbersome and hence harder to move from one site to another as the fairground moved around the country from one town to the next. As such, in the opening years of the 20th century, moving pictures started to switch from itinerant attractions that would have been encountered on the fairground to static displays. In fact, during this period, films were increasingly shown in vacant shops or pubs (see J. Barnes, 1983). However, the latter venues typically offered poor ventilation, and low air quality, as compared with what one might have expected to find in the presumably drafty fairground tent. The first purpose-built movie theaters were apparently not much better in terms of their ventilation either. Unsurprisingly, complaints about the smell of the great unwashed masses started to increase at this time. The stench would presumably have been especially noticeable in enclosed spaces, where many people were gathered together in close proximity, such as in the setting of popular early cinema screenings.

What is more, the problem of malodor was apparently much worse in movie theaters than in playhouses/theaters. In part, this was because the former would have been operating for longer each day (c. 7 hours) as compared with just a single show of 2 to 3 hours in the case of the theater—this, at least, according to a report from Richter (1926), a German engineer. Making matters worse, movie audiences may well have gone straight from work to the cinema (i.e., without having time to change). By contrast, those going out to the theater would have been far more likely to get dressed up specially for the occasion.^4^ On the basis of his calculations, Richter argued that theatergoers would likely have enjoyed 3 to 4 times more air per person than those in the cinema. It is worth noting that fragrant fountains in the lobbies, and program fans were also, occasionally a feature of theatrical performances in the latter half of the 19th century in London (e.g., see “Fan—Rimmel’s programme fan”, n.d.), again potentially helping to tackle the problem of malodor (see also Reinarz, 2003).

At the 1913 opening of Marmorhaus cinema in Berlin, the fragrance of Marguerite Carré, a perfume by Bourjois, Paris wafted through the building (Berg-Ganschow & Jacobsen, 1987). Given the timing, this is presumably more likely to have been a not so subtle attempt to mask the malodor of the masses rather than an early attempt to give a venue its own signature scent (see Spence, 2020b).

The stench that would arise during movie performances became so unbearable that handheld devices were soon introduced to spray deodorant over the audience’s heads 2 or 3 times per screening. And when this failed to solve the problem, 10-minute “airing breaks” were introduced in a desperate bid to help clear the air (Payer, 2001). Going further, in the 1920s, at Berlin’s Ufa-Palast, an electronically driven flying balloon (a blimp-like device) was launched in the auditorium in order to spray fragrant cologne water during the intermissions. In fact, so widespread was the problem of malodor that one of the early deodorants, going by the name of *Perolin* (that had a naphthalene-like smell), came to be the characteristic scent that cinema-goers would associate with the movies in the opening decades of the 20th century. The earliest examples of “sensehacking” cinema (Spence, 2021) were, then, all about the removal, or rather masking, of unpleasant olfactory sensations, rather than the addition of more senses (or new sensations) in order to enhance the experience (as tends to be the focus for those working on scenting the cinema today).

Nowadays, during the Covid pandemia lockdown, some people are even apparently craving the contemporary smell of the cinema (Feehan, 2020). Verissimo and Pereira (2013) conducted a study in a Portuguese cinema in Lisbon. A total of 407 moviegoers experienced a cinema complex that either had or had not been scented. The addition of scent was shown to positively impact people’s evaluation of the theater, the environment, and their intention to return when quizzed after watching the movie. Although the article itself is a little unclear, the ambient scent was of cola-lemon (though mint and popcorn aromas were also pretested).

## Early History of Synchronized (“Narrative”) Scents in the Cinema

The first “atmospheric” use of scent^5^ in a movie theater was by the cinema impresario S. L. (Samuel “Roxy”) Rothafel (1882–1936) in 1908. Rothafel ran the Rialto and Strand movie theaters in New York, as well as the eponymous Roxy, and was also the owner of a cinema in Forest City, Pennsylvania. It has been widely reported that he dipped cotton in rose scent, and held it in front of an electric fan at the latter venue, thereby suffusing the theater with floral fragrance during the newsreel screening of a Pasadena Rose Bowl Game (see Ijsselsteijn, 2003; Paterson, 2006; Ramsaye, 1926, p. 175). Although, as Gilbert (2008, pp. 148–149) notes, there was no Rose Bowl game in 1906, suggesting instead that the scented screening was more likely to have been associated with coverage of the New Year’s Day Rose Parade in Pasadena. In 1929, the manager of Boston’s Fenway Theatre added lilac perfume to the ventilation system designed to coincide with the movie’s title *Lilac Time* appearing on the screen (Gilbert, 2008, p. 149; Neale, 2008, p. 58). Meanwhile, an orange scent was released at Grauman’s Chinese Theatre in Los Angeles during the showing of MGM’s Hollywood Review. In the latter case, the smell was released during a big musical number called “Orange Blossom Time.”

In contrast to the low-tech scent delivery favored in the theater, cinema screenings gravitated toward a technical (and hence scalable) solution to the programmed delivery of scent instead. On March 4, 1930, John H. Leavell was awarded what appears to be the first patent in the United States pertaining to the automated delivery of scent in the cinema or theater (Leavell, 1930).^6^ Leavell’s suggested solution involved cutting notches in one side of the film to trigger a scent (such as the odor of the ocean to accompany an ocean scene) and to cut notches on both sides of the film should a second scent be required. (While this might be taken to suggest that the maximum number of scents that could have be triggered using this approach would have been two, the patent application itself talks about the possibility of delivering multiple odorants.) It is, though, worth stressing that an operator had to manually switch between the odour sources when multiple aromas were to be presented in a film.

The first film to incorporate multiple scents released sequentially to complement the events unfolding on the screen was shown at Paramount’s Rialto Theater on Broadway, New York by Arthur Mayer (1953, pp. 189–190). Gilbert (2008) suggests the unnamed inventor who promised synchronized scent for Mayer’s multisensory screening was none other than Leavell himself. Intriguingly, the scents that were mentioned included rose, honeysuckle, bacon, hospital smell (Lysol), car exhaust odor, and incense (see Wider & Aeppli, 1981).

On January 17, 1939, a patent was awarded to Melvin W. Merz, of Geneva, Illinois for an *Aroma diffusing apparatus* (Merz, 1939). According to the application:

This invention relates to an aroma diffusing apparatus and its general object is to provide an apparatus which is primarily designed for use in theatres and the like, such as motion picture theatres, for the purpose of releasing odors, to be spread throughout the theatre by air currents from a fan or cooling system in synchronized relation with a scene being shown and to be appropriate thereto or to correspond therewith, such as for example the odor of a perfume is spread when a ball room scene or scene of a lady appears on the screen, the scent or odor of exploding gun powder during a battle scene, the aroma of cooked foods during a restaurant scene, and etc., with the result it will be obvious that my aparatus [*sic*.] tends to make the pictures more realistic, than those at present.

Once again, notice how the focus here is on the pleonastic use of smells that are very literally, and hence redundantly, linked to the action on screen.

Merz (1939), though, did not seem to realize the challenges associated with distributing scent through a large space. The latter part of his patent application makes it sound as if this would somehow occur instantaneously. He writes:

It will be understood from the objects of this invention, that the apparatus which forms the subject matter thereof is arranged whereby the containers are disposed in the path of air currents from an electric fan or the air cooling system of the theatre, so that when the lids of the containers are opened, the odors will be spread throughout the entire area of the theatre, and of course the times of the opening of the lids are synchronized with certain scenes in the picture being shown, for the purpose and in the manner as set forth in the general object of the invention.

In 1939, Hans E. Laube, an advertising executive from Zürich started the “Odorated Talking Pictures” company together with financier Robert Barth and movie producer Conrad A. Schlaepfer. As a showcase for the new technology, the trio developed an English-language feature film by the name *My Dream* that included 20 scents. The system was unveiled at a press conference in Bern on December 2, 1939, garnering a mention in the *New York Times* (Crisler, 1940). The film was first shown in the United States at the Swiss Pavilion at the New York World Fair on October 19. According to Paech (n.d.), the system was capable of delivering up to 4,000 different scents. However, the equipment was soon confiscated by law enforcement agents for supposed patent infringement (Dumont, 1987, pp. 157–158),^7^ and according to Gilbert (2008), Laube never recovered it.

**Figure 1.**
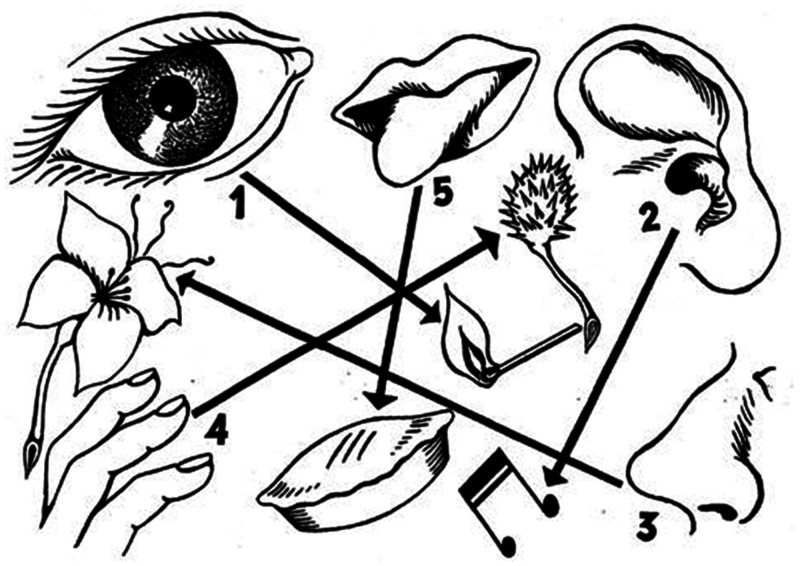
The order in which the senses focus attention according to Heilig (1955/1992, Figure 7).

A little over a decade later, Emery Imre Stern was awarded the U.S. patent for his smell-delivery system “Electromechanical scent distribution to accompany a motion picture” on August 7, 1951 (Stern, 1951a; see also Stern, 1951b). The application envisages the delivery of “a great variety of scents,” combined with a neutralizing agent, though, once again, it would appear that the system was never used in practice.^8^ Following the success of the introduction of “the talkies” (with Al Jolson in The Jazz Singer which came out in 1927 and was the first feature length film with not only music but lip-synchronized singing and speech), the sense of smell would seem to be the next most important in terms of capturing a cinema-goer’s attention. After all, according to Heilig (1955/1992), each sense monopolizes a person’s attention in the following proportions: sight 70%; hearing 20%; smell 5%; touch 4%; and taste 1% (see Figure 1). Notice here how smell is considered by Heilig as being more important even than touch. Heilig goes on to imagine a future for cinema in which: “The air will be filled with odors and up to the point of discretion or aesthetic function we will feel changes of temperature and the texture of things. We will feel physically and mentally transported into a new world” (p. 284).

In the patent awarded to Laube and Good (1957) on November 19, 1957, entitled *Motion pictures with synchronized odor emission,* the focus is on a system that is capable of delivering multiple odorants as well as the use of neutralizing agents. The application itself sketches out a case in which 25 odorant cells (for 15 distinct odorants with 10 repetitions) might be used in combination with three cells for an odor neutralizer giving rise to a total of 28 scent cells arranged on a rotating turntable. The application is very much centered on the question of how to deliver scents and then rapidly remove them. Eliminating the fixative in which odorants are usually mixed constituted part of the solution, together with the use of a neutralizing agent, and careful consideration of perceptual thresholds of the olfactory stimuli used (see also Laube, 1959).

### AromaRama

Building on Laube’s patents, in 1959, the “AromaRama” process was released by Charles (“Chuck”) Weiss, with smells piped-in through the air conditioning at the DeMille Theatre, New York.^9^ This approach to enhancing the olfactory element of cinema was first used in an Italian travelogue documentary film about the Great Wall of China called *Beyond the Great Wall* released on December 2. However, according to commentators, what ensued was “olfactory chaos” instead, with the scents rapidly becoming mixed, one with another (Crowther, 1959). It is easy to imagine how the air-conditioning systems were likely ill-prepared for the synchronized delivery, and thereafter the rapid removal, or neutralization, of a sequence of scents from the auditorium, especially a large space, as was often the case of early picture palaces (Nasaw, 1993; Richards, 2010). According to commentators writing at the time, the most successful of the scents was the initial burst of citrus that was released just as a juicy orange was sliced open and squeezed on screen in the prologue. The critics, however, complained about the temporal incongruency of audiovisual and olfactory elements that they found confusing. As *Time Magazine* (Anonymous, 1959) noted of the screening: “What is more, the smells are not always removed as rapidly as the scene requires: at one point, the audience distinctly smells grass in the middle of the Gobi desert” (p. 57). And, as if that was not enough, Crowther also criticized the scents as smelling synthetic, including the smell of cheap perfume, which Crowther took a particular dislike to. Crowther suggests that part of the problem here may well have been that the film was originally shot without any idea that it was to be accompanied by smells.

### Smell-O-Vision

The next year, January 12, 1960, saw the release of Mike Todd Jr’s version of Jack Cardiff’s thriller *Scent of Mystery,* starring the famous actress Elizabeth Taylor.^10^ The American producer took Laube’s process a stage further, renaming it *Glorious-Smell-O-Vision* under the auspices of their company Scentovison Inc. Clues to the identity of a murderer were provided by scents delivered direct to each seat. However, it is important to note that the experience was only ever available to audiences at a very small number of specially converted cinemas in New York, Chicago, and Los Angeles, where the seats were connected to a system of tubes linked to a central smell device. A signal on the film’s soundtrack was supposed to trigger the release of the appropriate scent in the auditorium. This was immediately followed by the release of a putatively neutralizing scent, prior to the release of the next smells in the sequence.

“The olfactory information,” writes Anne Paech, [a Constance-based film historian] “matched by and large the images on the screen, which *were connected with things such as garlic, gunpowder, wine, peppermint, shoe polish, lemons, fish, bananas, pipe tobacco, perfume and more than 20 other smells*” (quoted in Jütte, 2005, pp. 277–278).

However, despite all of the associated expense, this early state-of-the-art version of olfactory cinema was never anything more than a modest commercial success, according to Gilbert (2008) writing almost half a century later. Meanwhile, *The New York Times* film critic, Bosley Crowther (1960) wrote that: “If there is anything of lasting value to be learned from Michael Todd’s Scent of Mystery it is that motion pictures and synthetic smells do not mix.” Crowther, clearly not a fan of scented cinema, remember, was equally scathing of AromaRama. Some aromas delivered with a delay, others made people nauseas, and the smell machine apparently hissed loudly distracting from the film (Sebag-Montefiore, 2015).^11^ After the failure of the scented version, the film was soon renamed *Holiday in Spain* and marketed as a regular odorless movie. Thus, the outcome did not quite live up to Todd Jr’s optimistic proclamation that: “I hope it’s the kind of picture they call a scentsation!”

It is perhaps worth emphasizing here that Smell-O-Vision did not just have semantically congruent (and hence redundant) visual and olfactory information. Todd and Laube also used several crossmodal tricks to enhance the entertainment value of the scents that were released. So, for example, at one point, a taxi driver was shown drinking what looked like coffee while the audience smelled brandy instead (Sebag-Montefiore, 2015). Meanwhile, in another scene, a person slips at a market and the smell of ripe banana suggests the presumed cause, not shown on screen (slipping on a banana skin being a well-known visual gag). Finally, one of the characters in the movie is associated with a particular scent (namely, the tobacco smoke from actor Peter Lorre’s pipe), and when it is released later in the movie, it acts as a harbinger of the character’s reappearance on screen.

According to Gilbert (2008, p. 169), the organizers of the U.S. exhibit at the 1964 World’s Fair approached Laube about a scented movie project, but eventually dropped it. In subsequent years, several further patents were awarded for the synchronized delivery of odors with motion pictures. For example, in 1974, Westenholz et al. patented their *Apparatus for permeating an auditorium with odors in conjunction with projection of a motion picture film.* The focus in this case was on the effective removal of odorants once presented. However, it is unclear whether or not the proposed solution was ever taken up in a movie/commercial context. Another of the patents on the theme of synchronized scent delivery awarded to Götz-Ulrich Wittek was approved on November 3, 1998, under the title *Process and device for diffusing perfumes that accurately correspond to events or scenes during cinematographic representations and the like* (see Wittek, 1998). The latter application emphasized the way in which adding olfaction to moving images ought to serve to intensify the sensations associated with visual and acoustic representations (though provides no empirical evidence to support the claim plausible though it might be). What is noticeable about the latter application is the intention to deliver a variety of perfumes in close succession, as Wittek imagines:

John Malkovich kisses actress Debra Winger on the neck in a run-down hotel in Tangier in the movie “The Sheltering Sky,” and the audience Smells a bewildering blend of oriental perfume, the Sweet Scent of Skin and the basic odor of mould in the hotel room.

### Odorama: Scratch and Sniff: John Waters’ Polyester (1981)

In 1981, John Waters introduced *Odorama* with his movie *Polyester* featuring famous transvestite Divine (Waters, 1981). This low-tech solution involved scratch and sniff cards that had been impregnated with odors that were distributed to the members of the audience prior to the screening.^12^ The latter were supposed to scratch the corresponding spot on the card when the relevant number appeared on the corner of the big screen. There were a total of 10 smells including both pleasant scents, such as the smell of roses, new car smell, and pizza, and some really rather unpleasant smells, including the stench of flatulence and skunk (not to mention sweaty socks, or dirty tennis shoe, which was rated the worst by some; see deLahunta, 2003; Paterson, 2006; R. Williams, 1994). Members of the audience “enjoyed” a number of foul smells that were described in the film’s plot,^13^ which included the hallucinations of a glue sniffer. According to Waters, given his reputation as a director of trash movies, the decision to use bad smells, was supposed to render the experience less boring, and funnier.^14^

At the same time, however, it is also worth noting that recent virtual reality research has demonstrated how a person’s immersion in a digital experience tends to be enhanced more by the presence of bad smells than by either neutral or pleasant odors (see Baus & Bouchard, 2017). The latter simply just do not seem to do the same job. Indeed, there is now a growing body of research out there to suggest that the brain treats bad smells in an importantly different way from those smells that it finds pleasant or neutral. For instance, we respond significantly more rapidly to bad smells than to good ones (Boesveldt et al., 2010). What is more, we never seem to adapt to bad smells in quite the same way that we do to pleasant or neutral smells, such as the smell of our own home, either (Dalton & Wysocki, 1996; Spence, 2021). It might be argued, then, that Waters intuitively homed in on those scents, namely, unpleasant smells that were likely, in some sense, to be most effective.

### Interim Summary

Taken together, early high-tech solutions to scented cinema appeared to have failed because of problems with calibrating the intensity of scent delivery, problems with clearing, or neutralizing, the scent once released, and hence the ensuing problem of a lack of synchronization between what the audience was smelling at a particular moment and the action shown on the big screen (see also Levine & McBurney, 1986; McCartney, 1968; Vlahos, 2006). While the delivery of scent directly to each seat (in the case of Smell-O-Vision), rather than using the air-conditioning (as in the case of AromaRama), undoubtedly helped to address some of these problems, retrofitting cinemas for personalized scent release is likely to have been an expensive process. Low-tech scratch and sniff solutions proved slightly more successful (Waters, 1981), though, that being said, there can be little denying that the appeal of high-tech scent solutions remains amongst those writing in the press (Paterson, 2006). What is more, one of the problems with Odorama-type solutions, where viewers are given a hint to scratch by the numbers appearing on screen referencing which scent to scratch and sniff, risks taking people out of their engagement/immersion in the action, as John Waters himself recognized when talking to Avery Gilbert (2008): “I ask Waters if movie smells can be anything other than a gimmick? ‘You mean for real in a drama? No, I think it will always be a gimmick, because it takes you out of the movie’” (p. 167).

One of the other problems according to director Jack Cardiff when interviewed some decades after the release of *Scent of Mystery* was that the film failed because most of the scents smelled like “cheap cologne” (Sebag-Montefiore, 2015); this, remember, was also one of Crowther’s criticisms of the competing AromaRama system. R. Williams (1994) writes evocatively that at the end of one of these early scent-enabled movies: “the place smelt like a crowded brothel in a heatwave” (p. 69). If you are anything like me, then you will have to use your olfactory mental imagery to evoke this particular olfactory smellscape!

Another problem with the early use of scent in cinema is the sheer number of scents used. Initially, in 1939, Laube had suggested using no more than 10, because more would be “too much for the public’s nose” (see Gilbert, 2008, p. 164), but that number soon increased to 20, then, 30, and beyond. While on the one hand, this can be seen as making the most of what scent-technology had to offer, it also increases the risk of overloading the audience’s nostrils. For instance, 31 odors were sequentially released in the screening of *Beyond the Great Wall* and 30 in *Scent of Mystery* (Gilbert, 2008, p. 164). At the same time, however, as has been noted by commentators subsequently (Fujiwara, 2006), once the audience comes to expect scents, they may be disappointed if they are not presented—hence potentially leaving the film maker in something of an unfortunate “Catch-22” situation. Nevertheless, the striking difference with the seemingly more successful use of scent in the theater, is how few scents tend to be used there, and how often, the smells are literally of whatever happens to be on stage, be it a side of beef, or fireworks (Banes, 2001), or the actors cooking or smoking (Hawking, 2015; Margolies, 2003). In fact, according to Hawking (2015), scent is one of the least used senses in cinema.

It may be helpful here to consider the distinction that is drawn by Blankenship (2016) between the “atmospheric” and “narrative” use of scent. The former often involving a single constant scent more commonly used in the theater, at least traditionally. The narrative use of scent became more popular in the cinema setting once technological solutions to facilitate the delivery of multiple different scents started to become available.

A further problem for such technology-led smell delivery systems is that no one has been able to figure out a way of reducing odor perception to some number of odor primitives (as done so successfully in the case of color perception; see also Sebag-Montefiore, 2015). This is something that Heilig (1955/1992, p. 283) clearly failed to appreciate when writing early on that:

Odors will be reduced to basic qualities the way color is into primary colors. The intensity of these will be recorded on magnetic tape, which, in turn will control the release from vials into the theatre’s air conditioning system. In time all of the above elements will be recorded, mixed, and projected electronically—a reel of the cinema of the future being a roll of magnetic tape with a separate track for each sense material. With these problems solved it is easy to imagine the cinema of the future.

It is worth noting that familiar odors can have multiple meanings, or associations, for the audience. According to *Time Magazine* (Anonymous, 1959, p. 57), in the case of *Beyond the Great Wall* from 1958: “A beautiful old pine grove in Peking, for instance, smells rather like a subway rest room on disinfectant day.” Note that a similar “cleaning odor” response was also elicited in the theater setting more recently when, in a 2015 production of Sagittarius Ponderosa, a play by M. J. Kaufman, where a pine scent was released into the theater when the protagonist approaches a Ponderosa pine (see Blankenship, 2016). The inability to constrain the emotional association(s) that the audience had with scents, note, also a problem that stymied early attempts to bring the abstract use of scent to the theatrical or musical settings (e.g., Hartmann, 1913; Runciman, 1915). What is striking in the latter cases, though, is that in the cinema and theater, the visual information should have helped constrain the interpretation of the scent in a way that simply did not seem to happen (i.e., the cleaning scent interpretation won out, for at least some members of the audience, despite the visual backdrop).

## Recent History of Scent in the Cinema

Despite the early failures, there have been a number of attempts to scent the cinema since the heyday of interest in the latter half of the 20th century (e.g., Anonymous, 2006; Fujiwara, 2006; Sebag-Montefiore, 2015). However, what is especially noticeable is how such ventures typically constitute one-off novelties, seemingly designed more to attract media coverage by promoting the accompanying scent delivery, rather than necessarily a sustained attempt to bring scent to the cinema (in the way that color took over; see Bedi, 2017; Misek, 2010). Many of the latter attempts also reduced substantially the number of scents that were used. For instance, *Le Grand Bleu* (The Great Blueness), a film about diving was released in Paris, France, in 1989. At the moment that the blue sea appeared on screen, the tangy smell of sea salt started to pervade the auditorium. Once again, the air conditioning was used to distribute the scent. As described by Jütte (2005, p. 278), a single atmospheric scent was used in the movie. Other occasions when a single “atmospheric” scent has been introduced include the smell of chocolate for *Charlie and the Chocolate Factory*,^15^ and the smell of cut grass for screenings of *Gregory’s Girl* (as mentioned in Lwin & Morrin, 2012).

Patrick Suskind’s (1989) international bestselling novel *Perfume: The Story of a Murderer*, about a young man with incredible smelling abilities, was made into a movie that cried out for olfactory accompaniment (Jiaying, 2014; Sebag-Montefiore, 2015; Sobchack, 2013; Stone, 2007a). Somewhat ironically, given that film critics had applauded the way in which the original odorless version of the film managed to evoke the olfactory element that is key to the story (see Sebag-Montefiore, 2015), some could not resist the opportunity of adding scent. For instance, in one unique trial in Europe, in 2006, the audiences were handed scent blotters carrying the smell of urine and greasy hair to match aspects of the storyline (M. Harris, 2008).^16^

 Elsewhere, in 2006, one cinema in Tokyo and another in Osaka (Japan) were prepared for an olfactorily enhanced version of Terrence Malick’s 2005 movie *The New World,* starring Colin Farrell and Christian Bale, developed together with Japan’s NTT Communication Corporation. The idea here was to use scent to try and induce emotion and mood with devices placed below premium aroma seats in the last three rows of each cinema (in what was known as the “Premium Aroma zone”). These were all under computer control, and the scent was released according to a network-server-controlled timetable (Anonymous, 2006; Fujiwara, 2006; Herz, 2007, pp. 230–231). In this case, the program exhorted the audience to “enjoy a beautiful love story together with aromatic scents” (Fujiwara, 2006). Woodsy smells accompanied the arrival of the English in America, citrus infused the scene at the English court, while predominantly minty perfumes targeted the romantic scenes between Pocahontas and Captain John Smith. One problem with scented movies that film critic Chris Fujiwara (2006) has drawn attention to is that having been conditioned to expect there to be matching scents for what was shown on-screen, audiences were apt to be disappointed when they were not presented (so when viewing boiling leather, gunpowder, or when the Pocahontas character smells the pages of a book)—“the smell-sensitized viewer felt acutely the lack of a sympathetic aroma in the theatre” (see also Herz, 2007, p. 231).

Fujiwara (2006) concludes that:

On the whole, the experience was like watching a movie while an aromatherapy clinic was being held in the lobby. Even in my Premium Aroma Seat, I had a hard time distinguishing the scents and often was unsure if a new perfume were being introduced or if a random atmospheric shift had brought a residual scent into stronger focus.

Herz (2007, p. 231), meanwhile, had a somewhat more positive take, appreciating the pairing of scents with emotional scenes rather than specific visual stimuli, as has typically been used previously. These limited Japanese screenings are perhaps the only occasion where the cinema-goers were split into those who did, or did not, have the scent accompaniment. It is, though, unclear whether those with access to the scent had to pay more for their tickets or not, and, if so, what the price premium was.

The 2003 feature length cartoon *Rugrats go Wild!* was also distributed with Odorama scratch-and-sniff cards (Paterson, 2006). However, as Gilbert (2008, p. 166) notes, Waters’ lawyers at New Line Cinema succeeded in making Rugrats, Nickelodeon, and Viacom drop their use of the term “Odorama.” In 2011, Robert Rodriguez came out with *Spy Kids 4*, a 4D movie that was shot in Aroma-Scope (i.e., using scratch and sniff cards; Thill, 2011).

In 2015, *Scent of Mystery* returned briefly to screens in Bradford (United Kingdom) and Denmark (https://artandolfaction.com/projects/scent-of-mystery/; Sebag-Montefiore, 2015). To make the event a little more participatory (and presumably help keep the costs down), members of the audience were issued with fans that had been impregnated with the fragrance of the heroine. Heavily scented actors then moved around the auditorium, while other fragrances were released from spray bottles at the appropriate points in the film. It is though worth stressing that the aim of the revival is just that “It’s an opportunity to revive an interest in scented cinema.” according to producer and writer Tammy Burnstock (Sebag-Montefiore, 2015). Authentic aromas were used, and the total number of scents was halved as compared with the original. Nevertheless, even with these concessions, staging the scented version of this classic movie was apparently still an expensive undertaking.

### Edible Cinema: Mixed Reality Solutions

In recent years, there has been growing interest in those cinematic events where the audience gets to experience tasty (and often aromatic) snacks at various points in the film.^17^ For instance, Edible Cinema (https://www.ediblecinema.co.uk/) arranged a screening of the movie *Perfume* in the United Kingdom with a box of tasty treats to accompany it (Jamieson, 2012). Typically, numbered cards are held up by attendants in front of the screen in order to indicate when the audience should consume each of the numbered treats. While such foods are likely to be enjoyed retronasally rather than orthonasally (see Rozin, 1982), the sense of smell (olfaction) is nevertheless still involved (Jamieson, 2012; see Spence, 2015, for a review). For instance, in a screening of *Pan’s Labyrinth* by Edible Cinema, the audience were treated to burnt woody aromas of pine-scented popcorn for a scene at the start of the movie where the protagonist Ofélia is transported through the forest to her new home (Jamieson, 2012). Seven other consumable elements were included in this particular screening. I was involved in a similar series of screenings in the Everyman Screen on the Green cinema in London’s Islington a few years ago. Working together with artist Caroline Hobkinson and her team, small boxes of edible snacks were created to compliment screenings of the films *Gravity* and *X-Men: Days of Future Past* (see Anonymous, 2015). While the feedback from such events appears to be largely positive, I am not aware of anyone having compared cinema-goers’ enjoyment for those screenings with, versus without, the boxes of edible goodies. Although, as Ruth Jamieson worries, there is a danger that: “but is scrabbling around for snacks just a distraction?”

### Interim Summary

While scent-enhanced cinema is certainly not dead, it is far from a regular occurrence, and primarily seems to be introduced as a means of attracting media attention rather than to really enhance the multisensory viewing experience in the long term. It is typically more of a technical gimmick rather than anything else. What one needs to ask is why they have not been more successful. While the failure of certain early productions (e.g., *The Scent of Mystery*) has been put down by some commentators to the flimsy plot (Crowther, 1960; Sebag-Montefiore, 2015), scent has by now been added to enough films that one has to ask why it has never really caught on. On the one hand, it is easy to see how, if a film is specifically developed in anticipation of a scent accompaniment, then longer scenes (i.e., a bit like an act in a play) might help to deliver the scent and ensure that it had dispersed by the time the action cuts to the next scene (not withstanding any likely olfactory habituation effects, see later), given that this has often been commented on as a problem. While the technical challenges are certainly nothing to be sniffed at (as it were), it has also been argued that the psychological factors may have been a more important limitation to the appreciation of scent in cinema.

## Psychological Problems With Scented Cinema

While commentators have typically focused on *technological* problems limiting the widespread uptake of scent in the cinema, there are a number of *psychological* limitations that need to be born in mind, and which may, in fact, be equally important. One other factor to be aware of is that people have been shown to become functionally anosmic to the presence of ambient scents if their visual attention is distracted by a high-load task (Forster & Spence, 2018). One might think that an engaging movie is likely to be similarly demanding of a cinemagoer’s visual attention, hence meaning they might miss certain scents that would normally be perceptible. One can hardly expect people to pay a premium for an olfactory accompaniment that they may often not even be aware of. And, over and above any effects of attention, one should also consider the effect of habituation/cross-habituation (e.g., Epstein et al., 2005; Gottfried, 2006). It may be tempting to suggest that audiences might be left experiencing olfactory white after exposure to so many different smells (see Weiss et al., 2016). However, it should be remembered that the latter phenomenon has only been documented to occur under a very specific subset of conditions thus far. In particular, when 30 or more equi-intense odorants spanning the whole of odor space are presented. It is unlikely that such conditions will be experienced by accident in the context of the cinema.

Spence et al. (2017) have also drawn attention to what they have termed the fundamental misattribution error. This is the name given to the fact that even if it could be shown that a viewer’s enjoyment is significantly enhanced by the addition of scent, people are likely to attribute their pleasure to the action they see on-screen, given that we are all visually dominant (Gallace et al., 2012; Heilig,1955/1992; Hutmacher, 2019). Given the cost associated with preparing the relevant scents, not to mention (in some cases) refitting cinemas for scent delivery, the viewing public are unlikely going to be willing to pay for an experience unless they attribute it, rightly or wrongly, to the presence of scent. Given the existence of inattentional anosmia, and the possibly related fundamental misattribution error, it becomes clear how more is demanded of olfaction than was demanded of color, say, when it was first introduced to B&W movies in the 1930s. This may, in part, then help to explain why color movies have been so much more successful than scented cinema.^18^

Another relevant psychological factor concerns the fact that while watching a movie tends to be a very social activity, inasmuch as people like to talk about what they have seen, the fact that we all struggle to both imagine and describe smells in words means that such shared discussion is likely to be missing (Arshamian & Larsson, 2014; Majid & Burenhult, 2014; Yeshurun & Sobel, 2010).

## The Chemical Sensing of the Audience

According to Heilig (1955/1992):

Man is not only a “rational animal” but a “social animal,” as well, and just as he still gets dressed up and goes to a concert hall to hear music he could hear on the radio, he will continue to go to the neighbourhood movies to see the same film he could see at home on TV. (p. 291)

Could it be that part of the experience of communal cinema comes from smelling (even if unconsciously) the chemical signals given off by the other members of the audience? Researchers working in Mainz, Germany assessed the volatile chemicals that were present in the airflow vented out of a cinema seating 250 people (J. Williams et al., 2016). They collected data from more than 9,500 cinema-goers watching one of 108 screenings of 16 different films (such as *Hunger Games 2*). The results showed a significant increase in what the authors describe as “audience emitted chemicals” associated with thrill and comedy scenes. They measured chemicals given off from skin and from breathing, including carbon dioxide and isoprene (C_5_H_8_). These researchers detected changes in the chemical composition of the atmosphere that could be linked to the on screen action. What these results hint at is while the perceptible malodor of other bodies once distracted from the entertainment for those watching early cinema, subliminally perceived human chemosignals from the other members of the audience may, in fact, contribute subtly to enhancing the viewer’s multisensory viewing experience after all (see also de Groot et al., 2015; Golland et al., 2014).

It may not be going too far to suggest that people are sensitive to, albeit unconsciously, volatiles from the other members of the audience, and this, in some small way, contributes to what people appreciate about a shared experience (just as long as they are not too malodorous).^19^ According to J. Williams et al. (2016): “the chemical accompaniment generated by the audience has the potential to alter the viewer’s perception of a film” (p. 7). Here, it is worth noting that elsewhere researchers have reported that people are significantly better than chance at classifying whether t-shirts were worn by someone who had watched a funny versus fearful movie (Ackerl et al., 2002; cf. Chen et al., 2006), thus suggesting that it may be possible to isolate the relevant odorants to accompany specific emotions or moods.

## Conclusions

Long before anyone had thought about adding positive scents to match what was going on in the movie, the question of how to neutralize the bad smells associated with the presence of so many other customers that was the number one problem. Thus, from the very earliest days of moving pictures, there has been concern/interest around the olfactory contribution to the experience of film (Heilig,1955/1992). However, that interest was initially around how to eliminate the smell of the masses, it has since morphed into the enhancing of multisensory experience by engaging more senses.^20^ However, the failure of scented cinema to take off, unlike the phenomenal success of color in movies (e.g., Bedi, 2017; Misek, 2010), is often put down to problems with the technology (see Gilbert, 2008). And indeed technological challenges are especially noticeable in the context of the movie theater (e.g., as compared with the theater; see Banes, 2001). While problems associated with releasing, and then clearing, a sequence of scents in a theatrical setting have been around for more than a century (e.g., Hartmann, 1913; Shepherd-Barr, 1999), the use of scent in theatrical and music settings would seem to have more chance of succeeding both because a smaller number of scents have typically been involved, and also because the low-tech manual administration of scent is much more common (therefore lowering the cost) than the specially wired cinema seats that have, on occasion, been introduced (albeit only into a small number of cinemas). It is worth noting that the incorporation of scent has also been more successful in the context of the theme parks such as the Epcott center (Lukas, 2008), where the films are often specially commissioned specifically for the venue, and hence change much less frequently than the feature films that are typically shown in the cinema.

 Returning to the filmmaker, John Waters’ earlier comment that scent in cinema can only ever be gimmicky, Saskia Wilson-Brown, founder of LA’s Institute of Art and Olfaction and a collaborator on the 2015 showings of *Scent of Mystery* suggests that: “How do you make scent a part of the story without being gimmicky? It’s a question of semantics and creating a common language and common meaning, which is devilishly hard.” Note here also too that Crowther (1959) talked of scent in original showing of *Scent of Mystery* as being nothing more than a “stunt.” One can go for an atmospheric use of scent, but then that does not seem to make the most of what the technology has to offer once one has gone to all the trouble (not to mention expense) of installing it.

It can be argued that the use of scents needs to go beyond the mere redundant presentation of smells associated with that which can be seen (even though this was undoubtedly the focus of a number of the early patents in scent-enabled cinema) if it is to succeed in the long term (see Alberge, 2015). It is not merely enough to smell what one sees (in a redundant multisensory manner), but rather, olfactory cues need to provide something extra, be it a symbolic role (Banes, 2001), jokes (Waters, 1981), and so on. It is also worth noting that bad smells are likely to be more effective than positive scents in terms of their ability to enhance immersion (Baus & Bouchard, 2017). Low-tech scratch and sniff solutions, while easier to implement, risk taking the audience out of the film (Gilbert, 2008). Whereas automatically delivered may not be noticed, due to the phenomenon of inattentional anosmia (Forster & Spence, 2018). And, even if they are consciously perceived by the audience, the latter are likely to attribute their pleasure to what they see on screen, rather than to the presence of the scent(s), this what has been referred to as the fundamental misattribution error (Spence et al., 2017). Furthermore, the fact that people seem unwilling to pay much of a premium for scented cinema means that many of the scents that have been used to date have tended to come across as both cheap and synthetic, further limiting the appeal of this particular form of olfactory augmentation.

Nevertheless, looking to the future, there would appear to be a resurgence of interest in 4D cinema that incorporates scent as part of the enhanced multisensory experience (e.g., B. Barnes, 2014; Hall, 2018). According to one recent press report: “The new 4DX cinemas have been launched after smell was revealed to be the sense British movie fans would most like to have heightened when watching a film” (Hall, 2018). This renaissance supports the suggestion that:

“Commercial cinema,” notes the American film historian Patricia Mellenkamp, “bombards the senses of touch, taste and smell, as well as sight and hearing.” Yet this does not necessarily mean that we are moving towards new “sensuous” or even synaesthetic cinematic experiences. (Jütte, 2005, p. 278; Paech, n.d., p. 5)

When taken together with the atmospheric use of individual scents, scratch and sniff, and edible cinema-type solutions, there would seem to be a continued interest, albeit niche, in the incorporation of scent into the cinema.

Furthermore, as more content is viewed at home, what with the rise of services such as Netflix, not to mention the rise in new releases being streamed direct to the home in the era of pandemia, and so on, one might question how/whether olfactory scent delivery will increasingly make it into the home setting.^21^ Related to this, virtual reality, gaming, and porn industries have all talked about delivering on olfactory solutions for their respective audiences, be it on home entertainment systems, smart phones, and bespoke hardware (e.g., Cole, 2017; Kerruish, 2019; Natividad, 2016; Tan, 2012; Twilley, 2016; see also Pells, 2015; Ranasinghe et al., 2019; http://cartoonnetwork.com/promotion/smellytelly/; Spence et al., 2017, for a review). At the same time, however, it should be remembered that home-scented cinema was the dream of the Digiscents company. The latter even previewed The Wizard of Oz with scented accompaniment for journalists but note that dedicated scent delivery solution needed for each film given no one has yet figured out what the odor primitives might be (Dusi, 2014; see Spence et al., 2017, for a critical review).

Back in 1999, a journalist from *Wired* magazine talked of the Digiscents *i*Smell system (Platt, 1999). Delivering scented movie clips from *The Wizard of Oz*, with the aroma of cedar as Dorothy and her companions enter the forest, and the scent of wood smoke as the witch stirred her potion over a fire.^22^ However, one of the key problems for such systems is that no one has been able to figure out a way of reducing odor perception to some number of odor primitives. In a way, then, that brings us back to the same problem that prevented scented cinema from taking off. Just take the following quote to illustrate the point:

Turin believes that Smell-O-Vision has never taken off because, unlike colour TV, smell has no primaries that can be mixed to make endless combinations. “You cannot create an enormous palate of smells the way you can with [just three primary] colours,” he explains. “And that is a fundamental technological problem.” (quoted in Sebag-Montefiore, 2015)

## Notes

1 Heilig (1955/1992, p. 279) opens his article on the cinema of the future, originally published in 1955, by writing: “Pandemonium reigns supreme in the film industry. Every studio is hastily converting to its own “revolutionary” system—Cinerama, Colorama, Panoramic Screen, Cinemascope, Three-D, and Stereophonic Sound. A dozen marquees in Time Square are luring customers into the realm of a “sensational new experience.””

2 According to Heilig (1995/1992): “It is the addition of sound that represents the really great ‘revolution’ in the history of cinema” (p. 283). It is, though, worth noting that there was quite some resistance in the early days to the idea of “the talkies” (e.g., see Frost, 2006). And when it was eventually introduced, commentators were initially far from convinced that adding color would necessarily deliver as much of a step-change to the cinema-goers’ experience as adding a new sense, sound, had done a few years earlier (Anonymous, 1934).

3 My Dream, was screened at the New York World Fair/Exhibition in 1940. The film’s title referred to a ladies’ perfume that was popular at the time.

4 The tendency of men to wear gardenias in their lapels when going to the theater (see Sebag-Montefiore, 2016) would presumably also have helped to make the olfactory atmosphere in the theater a little more pleasant than in the cinema.

5 See Blankenship (2016) on the difference between the “atmospheric” and “narrative” use of scent in the theater.

6 According to the patent application: “It is an object of my invention to provide a novel method of presenting a theatrical production by which the olfactory sense, or the sense of smell, may be utilized in connection with other senses to communicate a complex theatrical impression to the patrons of a theater. It is a further object to provide a method of and apparatus for presenting a moving picture in which an odor will be presented to the sense of Smell of those viewing the picture which will awaken olfactory images closely associated with the visual images of the picture, thus strengthening the impression of reality made by the picture.” Notice the stress on the pleonastic use of scent to create congruent multisensory experiences of what is shown on screen. According to an anonymous reviewer of this study (with some experience of writing patents), the latter focus on the pleonastic use of scent may also reflect the way in which patents tend to be written.

7 One can only imagine that the infringement must have related to either Leavell’s (1930) or, perhaps more likely, Merz’s (1939) patent.

8 One other mention of the smellies being developed came from the Union of Soviet Socialist Republics. According to Gilbert (2008): “The Soviet Union, sensing another Cold War technology challenge from the Americans, tried to get in on the act. The Russian movie director Grigory Alexandrov claiming in 1949 that the Soviet film industry ‘was on the verge of producing smellies’, but there is no record they ever did” (p. 152)

9 It is worth noting that, in his early review, Morton Heilig (1955/1992) also suggests the release of scent programmed on magnetic tracks via the movie theater’s air conditioning.

10 At one time, Taylor had been married to Todd’s father, Michael (Gilbert, 2008). The Schiaparelli company apparently also produced a limited edition Scent of Mystery perfume, as worn by Taylor in the film (Gilbert, 2008, p. 158).

11 Bruce Kimmel remembered as a 67-year-old when first seeing, and smelling this movie in 1960 as a 12-year-old, the opening scent when a butterfly landed on a peach tree and the scent was released throughout the auditorium (Sebag-Montefiore, 2015).

12 According to Fujiwara (2006), the cards warned the members of the audience “Do not scratch until you receive instructions from the film.”

13 According to Jütte (2005), “The nauseating smells and brutal scenes of Water’s ‘shocker’ apparently turned the stomachs of some of the more sensitive spectators” (p. 278).

14 This from an interview with the director that appears in Gilbert (2008, p. 166).

15 This, according to Hall (2018), is apparently the film that cinema-goers would most like to smell.

16 The international fragrance company International Flavors & Fragrances (IFF) released a $700 collection of 15 scents by Thierry Mugler, designed as an olfactory interpretation of the novel Perfume. After smelling them all, one journalist was moved to write that “The final products, created by Christophe Laudamiel, and Christoph Hornetz, are a stunning and at times frightening example of perfume as art. They are about smelling in its purest sense and about evoking a time or a place through fragrance” (Stone, 2007b).

17 While food has long been integral to a trip to the movies (Maher, 2012), what is different about the secret cinema type events is that the flavours of the foods are linked in some way to the action on screen, hence are presumably less likely to distract (see Topolinski et al., 2013), at least if done well. That said, the dangers of such distracted eating leading to overconsumption are never far away (Zhou et al., 2017).

18 Van Toller (1988) makes an even more explicit comparison, writing that: “the sense of smell can be likened to colour in a television picture, it adds a tremendous amount in terms of texture, dimensionality, pleasure and feeling” (p. 184).

19 This, of course, something that is likely to be reduced, if not lost altogether, when social distancing measures are introduced in cinemas, as was done in the certain U.S. cinemas in the aftermath of the 1919 influenza outbreak (Arce, 1979, p. 103; Nasaw, 1993, p. 234), and as is being repeated almost exactly a century later with the current Covid-19 pandemic (though see also Morrison, 2020).

20 As German media specialist Anne Paech has been quoted as saying: “In its pursuit of the ultimate illusion, cinema has already made several serious attempts to heighten certain visual effects for the spectator by introducing corresponding olfactory sensations” (Jütte, 2005, pp. 277–278).

21 There was even a recent patent filed by Ford Motors to show films from the boot of one’s car (see Bridge, 2019).

22 It is worth pointing out, though, that Digiscents folded in 2001 when no hardware vendors materialized (see Dusi, 2014).
